# Metastatic Renal Cell Carcinoma versus Pancreatic Neuroendocrine Tumor in von Hippel-Lindau Disease: Treatment with Interleukin-2

**DOI:** 10.1100/tsw.2005.5

**Published:** 2005-01-14

**Authors:** Christopher Williams, McClellan Walther

**Affiliations:** Urologic Oncology Branch, Center for Cancer Research, National Cancer Institute, Bethesda, Maryland 20892, USA

**Keywords:** von Hippel-Lindau, neuroendocrine tumor, pancreas, renal cell carcinoma, metastases

## Abstract

Differentiating between clear cell neuroendocrine tumor (NET) of the pancreas and renal cell carcinoma (RCC) metastatic to the pancreas can be challenging in patients with von Hippel-Lindau disease (VHL). The clear cell features of both NET and RCC in VHL patients may lead to misdiagnosis, inaccurate staging, and alternative treatment. We present a patient in which this occurred. As clear cell NETs closely resembling metastatic RCC are distinctive neoplasms of VHL and metastatic RCC to the pancreas in the VHL population is rare, careful pathologic examination should be performed prior to subjecting patients to definitive surgical or medical therapies.

## INTRODUCTION

Differentiating between a clear cell neuroendocrine tumor (NET) of the pancreas and a renal cell carcinoma (RCC) metastatic to the pancreas can be challenging in patients with von Hippel-Lindau disease (VHL). The clear cell features of both NET and RCC in VHL patients may lead to misdiagnosis, inaccurate staging, and alternative treatment. We present a patient in which this occurred.

## CASE PRESENTATION

A 37-year-old, white male was referred to our institution for evaluation of manifestations of VHL. His complicated history prior to presentation at our institution included a partial nephrectomy for RCC and Interleukin-2 (IL-2) administration 10 months later for treatment of a pancreatic mass thought to represent a solitary metastatic RCC. IL-2 administration was preceded by an unsuccessful attempt at pancreatic biopsy. Subsequent to IL-2 treatment, the patient underwent laparoscopic resection of the pancreatic mass. The final pathology revealed a pancreatic NET, not metastatic RCC.

## DISCUSSION

VHL is an autosomal dominant disorder characterized by tumors and/or cysts of the pancreas, CNS, retina, kidney, adrenal gland, endolymphatic sac, epididymis, and broad ligament. The RCCs occurring in VHL are of clear cell histology and occur in approximately 24–45% of affected individuals[1]. Pancreatic manifestations of VHL include cysts, serous cystadenomas, and neuroendocrine tumors. Cysts are most common, occurring in approximately 14%, whereas, NETs occur in approximately 12% of affected patients[2]. NETs are solid, potentially malignant tumors composed of clear cells arranged in nests, cords, and tubules, similar to clear cell RCC. In comparison, metastatic RCC to the pancreas with VHL is rare.

Hoang et al. reported five VHL patients with clear cell pancreatic NETs that mimicked metastatic RCCs, two of which were confused with RCC on final diagnosis[3]. In comparison, 29 pancreatic neuroendocrine tumors not associated with VHL were evaluated, only one of which was found to have clear cell histology. Eleven sporadic RCCs metastatic to the pancreas also were evaluated, none of which expressed reactivity for neuroendocrine markers (chromogranin and synaptophysin). Electron microscopic and immunohistochemical studies in the VHL patients revealed that the NETs expressed neurosecretory granules and neuroendocrine markers, respectively.

Although case reports of VHL-related RCC metastatic to the pancreas exist, we agree with Hoang et al. that it is likely that many synchronous and metachronous pancreatic NETs in VHL patients have been interpreted as metastases. As clear cell NETs closely resembling metastatic RCC are distinctive neoplasms of VHL and metastatic RCC to the pancreas in the VHL population is rare, careful pathologic examination should be performed prior to subjecting patients to definitive surgical or medical therapies.
